# Nanotechnology and Cancer Bioelectricity: Bridging the Gap Between Biology and Translational Medicine

**DOI:** 10.1002/advs.202304110

**Published:** 2023-11-20

**Authors:** Rosalia Moreddu

**Affiliations:** ^1^ Istituto Italiano di Tecnologia CCT@Morego Genoa 16163 Italy

**Keywords:** Bioelectricity, Cancer, Electrophysiology, Ion Channels, Nanotechnology, Non‐Excitable Cells, Translational Medicine

## Abstract

Bioelectricity is the electrical activity that occurs within living cells and tissues. This activity is critical for regulating homeostatic cellular function and communication, and disruptions of the same can lead to a variety of conditions, including cancer. Cancer cells are known to exhibit abnormal electrical properties compared to their healthy counterparts, and this has driven researchers to investigate the potential of harnessing bioelectricity as a tool in cancer diagnosis, prognosis, and treatment. In parallel, bioelectricity represents one of the means to gain fundamental insights on how electrical signals and charges play a role in cancer insurgence, growth, and progression. This review provides a comprehensive analysis of the literature in this field, addressing the fundamentals of bioelectricity in single cancer cells, cancer cell cohorts, and cancerous tissues. The emerging role of bioelectricity in cancer proliferation and metastasis is introduced. Based on the acknowledgement that this biological information is still hard to access due to the existing gap between biological findings and translational medicine, the latest advancements in the field of nanotechnologies for cellular electrophysiology are examined, as well as the most recent developments in micro‐ and nano‐devices for cancer diagnostics and therapy targeting bioelectricity.

## Graphical abstract

Bioelectricity regulates key life processes, including cell proliferation, migration, and mutation. Cancer cells are known to exhibit different electrical properties compared to their healthy counterparts. However, this information is yet to be fully unfolded due to the complexity of such signals. This review presents the potential that nanotechnology holds in providing the tools to understand and exploit these phenomena.

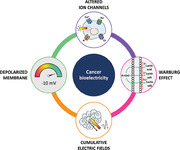

## 1 Introduction

**Figure 1:**
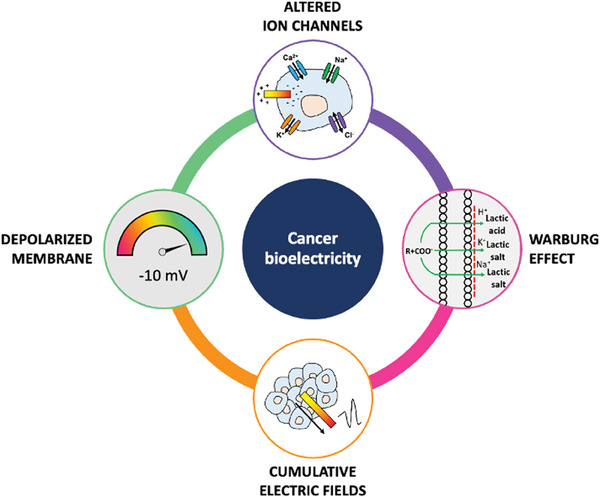
Overview of the multifactorial interplay in cancer bioelectricity.

Cancer is a complex disease that affects millions of people worldwide.^[^ ^1^ ^]^ Despite advances in cancer research and treatment, cancer remains one of the leading causes of death globally.^[^ ^2^ ^]^ In recent years, there has been a growing interest in exploring the role of bioelectricity in cancer.^[^ ^3^, ^4^, ^5^, ^6^ ^]^ This field of study aims at understanding how electrical signals and charges play a role in cancer insurgence, growth, and progression.^[^ ^7^ ^]^ This is based on the findings that cancer cells exhibit different electrical properties compared to healthy cells, and this difference can be used as a diagnostic and therapeutic tool.^[^ ^3^, ^8^, ^9^, ^10^ ^]^ A variety of techniques, including electrophysiological recording, impedance spectroscopy, and impedance imaging, revealed that these alterations result from the interplay of multiple interconnected factors (**Figure** **1**).^[^ ^5^, ^11^ ^]^ For example, cancer cells have a higher membrane impedance, which makes them less electrically conductive than normal cells.^[^ ^8^, ^12^ ^]^ This can affect the flow of electrical signals within the cell, leading to changes in cellular behavior, such as increased proliferation, reduced apoptosis, increased migration directionality, and invasion capabilities.^[^ ^13^, ^14^ ^]^ Bioelectricity is also gaining interest as a crucial phenomenon involved in the regulation of homeostatic functions in non‐excitable cells, including cell division, migration, and ageing.^[^ ^14^, ^15^ ^]^ Single cancer cells were reported to undergo the so‐called Warburg effect, yielding to the excretion of lactate anions that establish a negative surface charge on the outer layer of the cancer cell membrane.^[^ ^16^ ^]^ As a result of the electrical variations observed in single cells, cancer cell populations have also shown to exhibit bioelectric cohort effects, for example by establishing endogenous current gradients across portions of cell mono‐ or multi‐ layers and tissues.^[^ ^11^ ^]^

In parallel, emerging therapeutic approaches are based on the idea that electric fields can be used to disrupt the behavior of cancer cells, by affecting the activity of ion channels to induce selective apoptosis.^[^ ^17^, ^18^, ^19^, ^20^, ^21^ ^]^ These methods aim to overcome the side effects associated to traditional cancer treatments, such as chemotherapy and radiation therapy.^[^ ^22^, ^23^ ^]^ Complementary approaches based on bioelectricity have been also considered to enhance the effectiveness of existing therapeutic strategies. For instance, a recent field of research, known as electroceuticals, aims to use electrical signals to manipulate cellular behavior and induce therapeutic effects.^[^ ^5^ ^]^ Electroceuticals may also be used to enhance the immune response to cancer, by stimulating the immune system.^[^ ^16^, ^24^, ^25^ ^]^

According to the World Health Organization (WHO) latest report released in early 2023,^[^ ^1^ ^]^ cancer is responsible for ≈9.6 million deaths each year. In terms of incidence, the most common types of cancer vary depending on geographic location and other factors, such as age and lifestyle. Some of the most diagnosed types of cancer include lung cancer, breast cancer, colorectal cancer, prostate cancer, and stomach cancer.^[^ ^1^ ^]^ Breast cancer is the most common cancer among women worldwide, accounting for 25% of all cancer cases. Prostate cancer is the most common cancer in men, accounting for nearly 15% of all new cases. Lung and colorectal cancers are the second and third causes of cancer death in both genders, accounting for approximately the 18% and the 10% of all cases, respectively.^[^ ^1^ ^]^ In addition to its potential applications in cancer diagnosis and treatment, cancer bioelectricity also holds promise for improving our fundamental understanding of cancer biology.^[^ ^9^ ^]^ Bioelectricity has been recently investigated in subcellular structures, such as organelles^[^ ^26^ ^]^ and mitochondria.^[^ ^27^ ^]^

### 1.1 Brief History of Bioelectricity

The study of bioelectricity dates to the 18th century, when Italian physician Luigi Galvani discovered that the muscles of a dissected frog leg would twitch when touched with a charged metal object. He hypothesized that there was a type of electricity in living organisms, which he called *animal electricity*. Galvani's work was further developed by his nephew, Giovanni Aldini, who applied electrical stimulation to dissected human corpses, causing their limbs to move. In the early 19th century, German physiologist Emil du Bois‐Reymond conducted experiments on living animals and demonstrated the existence of electrical signals in nerves and muscles, which he named *action potentials*. In 1875, British physiologist Richard Caton discovered that the brains of animals produced electrical signals, which he recorded using a primitive device, today known as the galvanometer. This led to the development of the electroencephalogram (EEG) to record the electrical activity of the brain. In the early 20th century, the invention of the cathode ray oscilloscope allowed researchers to measure these electrical signals more precisely, leading to the invention of the electrocardiogram (ECG), which measures the electrical activity of the heart. In the 1930s, American physiologist Albert Szent‐Györgyi discovered that certain chemicals, such as adenosine triphosphate (ATP), played a role in the generation and regulation of electrical signals in living organisms. This led to the discovery of ion channels, which are transmembrane proteins that allow charged particles to pass through cell membranes, and the development of the Hodgkin‐Huxley model, which explains how ion channels generate action potentials in neurons.

**Figure 2:**
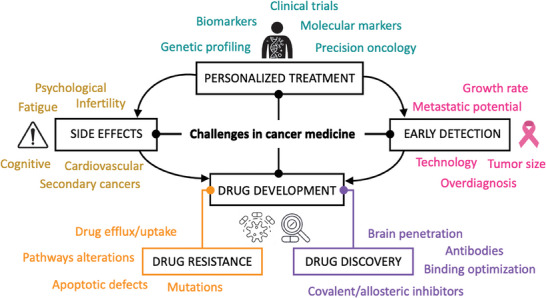
Current challenges in cancer medicine: early detection, personalized treatment, long‐term side effects, and drug development.

In the 1960s and 1970s, American biophysicist Andrew Huxley and British physiologist Alan Hodgkin were awarded the Nobel Prize in Physiology for their work on the mechanisms underlying the generation and propagation of action potentials in neurons. This work laid the foundation for the modern field of neurophysiology. Today, bioelectricity is a rapidly growing field of research that encompasses many areas, including the study of ion channels and other cellular components, such as mitochondria, that generate and regulate electrical signals, the use of electrical stimulation to treat neurological disorders and promote tissue regeneration, and the development of bioelectronic devices for diagnostic and therapeutic purposes. The field of cancer medicine is under constant development in terms of technological advances and fundamental understanding. At present, the main challenges in cancer medicine are early detection, personalized treatments, access to care, drug development, long‐term side effects, and regulatory landscape.^[^ ^19^, ^28^ ^]^ The main players are schematized in **Figure** **2**.

## 2 Electricity of Single Cancer Cells

**Figure 3:**
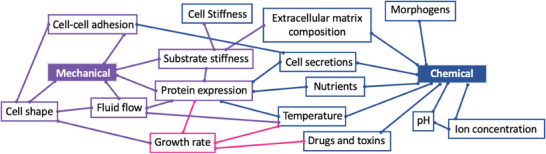
Chemical and mechanical cues associated to cancer cell bioelectricity.

Alterations in the bioelectric properties of individual cancer cells have been observed in various types of cancer.^[^ ^5^, ^9^, ^29^, ^30^ ^]^ For example, some studies have found that cancer cells exhibit depolarized membrane potentials, resulting from altered expression of ion channels or other signaling pathways.^[^ ^31^ ^]^ Depolarized membrane potentials can lead to increased calcium influx, which activates downstream signaling pathways that promote cancer cell growth and survival.^[^ ^14^ ^]^ In addition, some studies have found that the impedance of cancer cells can differ from that of normal cells.^[^ ^31^ ^]^ For example, cancer cells may exhibit increased membrane capacitance or decreased membrane resistance, which can reflect changes in membrane permeability or ion channel function.^[^ ^32^ ^]^ Cancer cell bioelectricity is strictly linked to mechanical and chemical cues (**Figure** **3**).^[^ ^13^ ^]^ Mechanical triggers include cell‐cell adhesion, cell shape, and protein expression. The latest contributes to dictate the growth rate of the cancer cell, which in turn is connected to temperature gradients, that are associated to chemical variations. Chemical cues include, among all, the composition of the extracellular matrix, pH and ion concentrations, and cell secretions.

Overall, the bioelectricity of cancer cells may be summarized by analyzing three main interconnected components: ionic currents, faradaic currents, and cell surface charges.^[^ ^9^ ^]^ Ionic currents result from the movement of charged ions, faradaic currents are the product of reduction and oxidation of biochemical molecules, and the Warburg effect is the cause of negative surface charges observed in cancer cells.^[^ ^12^ ^]^

### 2.1 Membrane Potential of Cancer Cells

**Figure 4:**
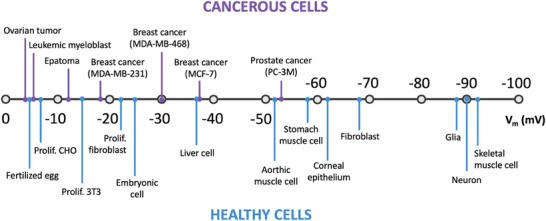
Membrane potential of selected cancerous and healthy cell types. Readapted from.^[^ ^14^ ^]^

The membrane potential V_m_ results from the uneven distribution of charged ions between the cytoplasm and the extracellular environment, and it is a key biophysical signal in non‐excitable cells that modulates fundamental biological mechanisms, such as cell proliferation and differentiation. It is defined by the Goldman–Hodgkin–Katz equation:^[^ ^33^, ^34^ ^]^

(1) $$V_{m}=\frac{RT}{F}\;ln\left(\frac{P_{Na^{+}}{\left[Na^{+}\right]}_{o}+P_{K^{+}}{\left[K^{+}\right]}_{o}+P_{Cl^{-}}{\left[Cl^{-}\right]}_{o}}{P_{Na^{+}}{\left[Na^{+}\right]}_{i}+P_{K^{+}}{\left[K^{+}\right]}_{i}+P_{Cl^{-}}{\left[Cl^{-}\right]}_{i}}\right)$$

where P is the permeability of the membrane to specific ions, *R* is the ideal gas constant, *T* the temperature, and *F* the Faraday constant. The subscripts *i* and *o* indicate the inside and the outside of the cell, namely the cytoplasm and the extracellular environment, respectively. V_m_ fluctuations are not only dictated by the cell itself, but also by its surrounding and cell‐cell communications, such as gap junctions. Electrophysiological analyses in many cancer cell types have revealed a depolarized V_m_ that favors cell proliferation.^[^ ^5^, ^14^ ^]^ **Figure** **4** depicts on a scale the average V_m_ values that have been experimentally measured in selected cancerous and healthy cells. **Table** **1** reports the average cancer cell V_m_ compared to its healthy counterpart, based on the body site.

**Table 1:** V_m_ alteration in selected healthy and cancerous cells collected from ref. [10].

Body site	V_m_ [healthy] mV	V_m_ [cancer] mV	V_m_ ratio [cancer/healthy] a‐dimensional
Brain	−70	−55	≈0.8
Breast	−37	−36	≈1
Immune system	−19	−7	≈0.4
Liver		−7	
Lung		−33	
Reproductive	−31	−17	≈0.5
Stomach	−45	−24	≈0.5
Thyroid	−38	−50	≈1.3

V_m_ regulation plays a role also in cell migration (e.g., wound healing),^[^ ^35^ ^]^ cancer progression, and metastasis.^[^ ^36^ ^]^ In cancer cells, V_m_ undergoes hyperpolarization before entering the M phase, associated to mitotic activity, suggesting that V_m_ is correlated to cell cycle progression and can reversibly block DNA synthesis and mitosis.^[^ ^14^ ^]^ In the context of cancer cell division, membrane depolarization might be important for the emergence and maintenance of cancer stem cells (CSCs), giving rise to sustained tumor growth.^[^ ^37^ ^]^ This aspect will be addressed in *Section* *4.1* of this review.

### 2.2 Ionic Currents

**Figure 5:**
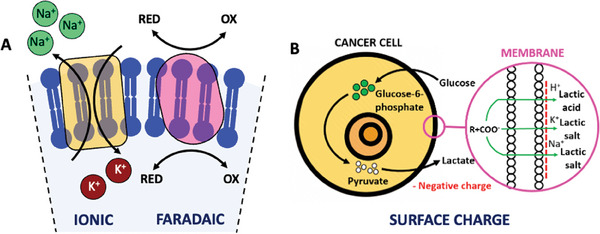
Bioelectrical phenomena observed in single cancer cells. A) Ionic currents and faradaic currents; B) Warburg effect in cancer cells: negatively charged surface profile.

All cells in the body constantly transport ions and other charged molecules across the phospholipidic membrane (**Figure** **5**). This transport occurs via ion pumps or ligand‐gated channels that respond to electrochemical gradients (Figure 5A). The accumulation of charged particles at both sides of the cellular membrane establishes a potential different between the cytoplasm and the extracellular matrix, commonly referred to as the membrane potential (V_m_).^[^ ^38^ ^]^Organelles, such as mitochondria, also possess membrane potential gradients.^[^ ^39^ ^]^ Transport and distribution of charged molecules also induce electrical effects at cellular, tissue, and organ levels, to trigger various signaling responses.^[^ ^9^ ^]^ **Table** **2** presents an overview of the main ion channels which are found to be dysregulated in cancer cells, for selected cancer types. The main ion channels that regulate cellular electricity are sodium, potassium, calcium, and chloride channels, which are individually addressed in the following subsections, with a focus on their role in cancer electricity.

**Table 2:** Altered ion channels in selected cancer types collected from ref. [6, 8, 43, 44, 45].

Cancer type	Altered ion channels			
	Na^+^ subtype	K^+^ subtype	Ca^2+^ subtype	Cl^−^ subtype
Breast	nNav1.5, nNav1.7	Kv1.3, Eag1, Kir2.1, Kir2.2, Kir2.3, Kir3.1, Kir3.2, Kir4.1, TASK1, TASK3, SK4	L‐type VGCCs, SOCE, TRPV6	
Colon	nNav1.5,	Kv1.3, Kv1.5, Eag1, SK4, HERG1	L‐type VGCCs	
Smooth muscle		Kv1.3		
Skeletal muscle		Kv1.3, Kv1.5		
Lymph node		Kv1.3, Kv1.5		
Glioblastoma		Kv1.3, Kv1.5, hERG1, SK4		CLC‐3
Leukaemia		Kv1.3, hERG1		
Pancreas		Kir2.1, Kir2.2, Kir2.3, Kir3.1, Kir3.2, Kir4.1		CLCA
Prostate	(n)Nav1.7, Nav1.6	Kir2.1, Kir2.2, Kir2.3, Kir3.1, Kir3.2, Kir4.1, TREK1, TREK2, SK4	TRPV6	
Nasopharyngeal				CLC‐3
Colorectal		SK4		CLCA
Lung	Nav1.7	TASK1, TASK3		
Mesothelioma	Nav1.2, Nav1.6, Nav1.7			
Cervical	nNav1.6			
Stomach	Nav1.7	Kv1.5		
Ovary	Nav1.5	TREK1, TREK2	TRPV6	CLCA
Melanoma	Nav1.5	hERG1		
Oral squamous cell	Nav1.5			
Neuroblastoma	nNav1.5, Nav1.7			
Endometrium	Nv1.7			

#### 2.2.1 Sodium Channels (Na^+^)

**Figure 6:**
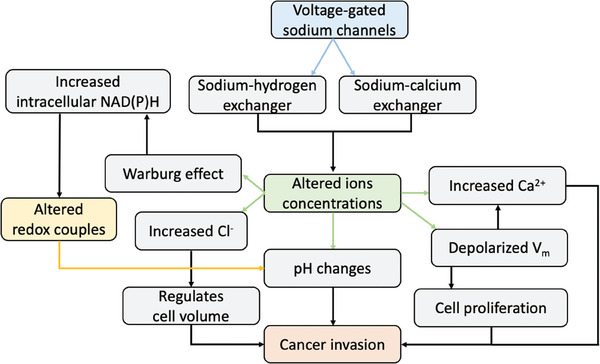
Cascade of events triggered by the alteration of voltage‐gated sodium channels in cancer cells.

Sodium channels are known to regulate the generation and propagation of action potentials in excitable cells.^[^ ^40^ ^]^ Recent research has also found that sodium channels are overexpressed in many types of cancer cells, and that their expression is correlated with more aggressive tumor behavior.^[^ ^41^ ^]^ **Figure** **6** schematizes the cascade of events associated with the presence of altered voltage‐gated sodium channels (VGSC) in cancer cells. The overexpression of sodium channels in cancer cells is thought to contribute to cancer progression in several ways: (i) *Increased cell proliferation*. Sodium channels promote cell proliferation and survival in cancer cells through the activation of downstream signaling pathways that promote cell growth and inhibit cell death.^[^ ^42^ ^]^

(ii) *Enhanced migration and invasion*. By regulating cytoskeletal dynamics, sodium channels activity can trigger cancer cells to migrate and invade surrounding tissues.^[^ ^36^ ^]^ (iii) *Tumor angiogenesis*. Angiogenesis, i.e., the formation of new blood vessels in tumors, can be promoted by altered sodium channels activity in cancer cells through the activation of signaling pathways that promote the release of pro‐angiogenic factors.^[^ ^46^ ^]^ Consequently, sodium channels suppression has been targeted as a potential therapeutic strategy.^[^ ^8^ ^]^ Local anesthetics and anti‐epileptic drugs have shown to have anti‐cancer effects in preclinical studies.^[^ ^47^ ^]^ Selected sodium channels that are altered in cancer cells, together with their inhibition effects, are presented in **Table** **3**.

**Table 3:** Selected sodium channel types, their inhibitors, and the effect of their altered expression in cancer cells.^[^ ^9^ ^]^

Na^+^ channel type	Na^+^ channel subtype	Inhibitors	Inhibition effect
Voltage‐gated (VGSC)	NaV1.5	Ropivacaine, ranolazine, TTX, propranolol, EPA	Regulates invasion and aggressiveness (colon, breast, and ovarian cancers)
Epithelial (ENaC)	ENaC.x		Drives sodium flux

#### 2.2.2 Calcium Channels (Ca^2+^)

Calcium channels are a family of transmembrane proteins that regulate the passage of calcium ions through cell membranes. They play important roles in various physiological processes, including muscle contraction, neurotransmitter release, and gene expression.^[^ ^48^ ^]^ In recent years, there has been growing evidence to suggest that calcium channels also play a role in cancer cell biology.^[^ ^44^ ^]^ Interestingly, calcium channels have also been recently identified in bacteria for the first time.^[^ ^49^ ^]^ **Table** **4** presents selected calcium channels and their effect on tumor development. Several types of calcium channels, including voltage‐gated calcium channels (VGCCs), store‐operated calcium channels (SOCs), and transient receptor potential (TRP) channels, are overexpressed in various types of cancer cells, and this has been correlated to increased cell proliferation, migration, invasion, and angiogenesis.^[^ ^44^ ^]^ (i) *Cell proliferation*. Calcium channels affect cell proliferation rate via the activation of signaling pathways that promote the expression of genes involved in cell cycle progression and DNA replication. For example, VGCCs were reported to promote cell proliferation in prostate cancer cells through the activation of the protein kinase C (PKC) pathway.^[^ ^50^ ^]^ Similarly, SOCs have been shown to promote cell proliferation in breast cancer cells through the activation of the nuclear factor of activated T cells (NFAT) pathway.^[^ ^51^ ^]^ (ii) *Cell migration and invasion*. Calcium channels have also been shown to promote cancer cell migration and invasion, through the regulation of cytoskeletal dynamics.^[^ ^52^ ^]^ For example, VGCCs were reported to promote cell migration and invasion in glioma cells through the activation of the RhoA/ROCK pathway.^[^ ^53^ ^]^ Similarly, TRP channels were shown to promote cell migration and invasion in breast cancer cells through the activation of the PI3K/Akt pathway.^[^ ^54^ ^]^ (iii) *Tumor angiogenesis*. Calcium channels have been implicated in the formation of new blood vessels in tumors. For example, SOCs have been shown to promote angiogenesis in melanoma cells through the activation of the hypoxia‐inducible factor (HIF) pathway,^[^ ^55^ ^]^ and TRP channels were reported to promote angiogenesis in glioma cells through the activation of the VEGF pathway.^[^ ^56^ ^]^ Anti‐cancer drug based on calcium channels inhibition were found to be successful in preclinical studies.^[^ ^57^ ^]^ For example, the calcium channel blocker verapamil inhibited tumor growth and angiogenesis in various types of cancer cells.^[^ ^43^ ^]^ Therefore, this approach may represent a promising avenue for the development of new cancer therapies.

**Table 4:** Selected calcium channels, their inhibitors, and the effect of their altered expression in cancer cells.^[^ ^9^ ^]^

Ca^2+^ channel type	Ca^2+^ channel subtype	Inhibitors	Inhibition effect
Voltage‐gated (VGCC)	L‐type VGCC T‐type VGCC	Amlodipine, diltiazem, verapamil N/A	Inhibits proliferation (HT‐39 breast cancer Promotes apoptosis (HCTT116 colon cancer)
Store‐operated calcium entry (SOCE)	N/A	N/A	Augmented expression of cyclin with reduced proliferation (breast cancer cells)
Transient receptor potential (TRP)	TRPV6	SOR‐C13	Downstreams invasion signaling (breast, ovarian, prostate cancers)

#### 2.2.3 Potassium Channels (K^+^)

Potassium channels regulate the passage of K^+^ ions across cell membranes. In healthy cells, K^+^ channels play a critical role in maintaining the resting membrane potential, regulating cellular excitability, and contributing to the cellular signaling required for normal physiological processes.^[^ ^58^ ^]^ However, in cancer cells, the expression and function of K^+^ channels was found to be altered, which may contribute to tumor growth and progression. Multiple types of K^+^ channels have been identified in cancer cells, including voltage‐gated K^+^ (K_v_) channels, calcium‐activated K^+^ (KCa) channels, and inwardly rectifying K^+^ (Kir) channels.^[^ ^59^ ^]^ These channels can be differentially expressed in various types of cancer, and the expression of specific K^+^ channels can be used to identify different cancer subtypes. **Table** **5** presents selected K^+^ channels and the effect of their expression in cancer cells. Alterations in K^+^ channel expression in cancer cells can result from genetic mutations, epigenetic modifications, or changes in the tumor microenvironment.^[^ ^60^ ^]^ For example, some studies have reported that hypoxia in the tumor microenvironment can lead to increased expression of K_v_ channels, which can promote cancer cell survival and proliferation.^[^ ^61^ ^]^ Several strategies are being explored also for targeting K^+^ channels in cancer. For example, some small molecule inhibitors that target K_v_ channels and antibodies that can selectively block the function of specific K_v_ channels yielded promising results in preclinical studies.^[^ ^59^ ^]^

**Table 5:** K^+^ channels and the effect of their altered expression in cancer cells.

K^+^ channel type	K^+^ channel subtype	Inhibitors	Inhibition effect	Ref.
Voltage‐gated (VGPC)	*Delayed rectifier (Kv)* Kv1.3	MgTX Piperine PAP‐1 Rituximab	*Induce apoptosis by arresting cell cycle in G1 phase* Blocks cell proliferation (in vivo adenocarcinoma) Decrease tumor volume (in vivo adenocarcinoma) Decreases proliferation (prostate cancer cells) Decreases proliferation (leukemia)	[62] [63] [9] [9] [64] [65]
	Kv1.5	4‐AP TEA	Affects T‐cells function Reduces cancer progression (glioma cancer cells)	[9]
	*Ether‐a‐go‐go (EAG)* Eag‐1 (also called Kv10.1) hERG1 (also called Kv11.1)	Imipramine, astemizole, tetrandrine E‐4031	*Major regulation of cancer progression* Reduces cancer progression (breast cancer cells) Decreases tumor growth (cervical, colon, breast cancers)	[66] [67]
Inward rectifiers (Kir)	Kir2.x family Kir3.2	Gambogic acid (GA) Piezoelectric nanoparticles	Reduces cell prolfieration (glioblastoma, leukemia, melanoma, colon cancer cells) Anti‐proliferative effect by pore blocking (breast, prostate, pancreas)	[9]
Two‐pore domain (K2P)	TREK1 TASK3	EGCC, curcumin, quercetin Tetrandrine, WFA	Inhibit proliferation by morphologica aberrations (breast cancer cells) Blocks proliferation, hallmark of stage (prostate and ovarian cancers) Regulate apoptosis (breast and lung cancers)	[68] [9]
Calcium‐activated (SK)	SK4	TAM+NS6180 TRAM‐34	Blocks migration and proliferation (glioblastoma, breast, prostate cancers) Reduces tumor size (glioma‐bearing mice in vivo)	[69] [9]

#### 2.2.4 Chloride Channels (Cl^−^)

Chloride ion channels are transmembrane proteins that play critical roles in a variety of physiological and pathological processes. In healthy cells, chloride channels help regulate cell volume, maintain pH balance, and control ion movement across the cell membrane. Recent studies have shown that the expression and activity of chloride ion channels are altered in cancer cells, leading to changes in cellular behavior and promoting tumor growth and metastasis.^[^ ^6^ ^]^ **Table** **6** presents selected chloride channels and their implication in tumor development. One of the most studied chloride ion channels in cancer is the cystic fibrosis transmembrane conductance regulator (CFTR), which is mutated in patients with cystic fibrosis. CFTR is a cAMP‐regulated chloride channel that is expressed in various epithelial tissues, including lungs, pancreas, and gastrointestinal tract.^[^ ^70^ ^]^ In cancer, CFTR expression and function are dysregulated, leading to alterations in cell proliferation, migration, and invasion. For example, studies have shown that CFTR is overexpressed in several types of cancer, including breast, colon, and lung cancer. In breast cancer, CFTR has been shown to promote cell proliferation and migration by activating the PI3K/Akt signaling pathway.^[^ ^71^ ^]^ In colon cancer, CFTR expression is associated with increased invasiveness and metastasis.^[^ ^72^ ^]^ Similarly, in lung cancer, CFTR overexpression is correlated with poor patient prognosis and increased tumor growth.^[^ ^73^ ^]^ Another channel that has been implicated in cancer is the chloride intracellular channel 1 (CLIC1).^[^ ^74^ ^]^ CLIC1 is a member of the glutathione S‐transferase (GST) superfamily and is known to regulate cell proliferation and survival. CLIC1 has been shown to promote cell proliferation and invasion in breast cancer by activating the Wnt/β‐catenin signaling pathway. In lung cancer, CLIC1 overexpression is associated with increased tumor growth and angiogenesis. Other chloride ion channels that have been studied include the volume‐regulated anion channel (VRAC), the Ca^2+^‐activated chloride channel (CaCC), and the voltage‐gated chloride channel (CLC).^[^ ^45^ ^]^ VRAC is involved in regulating cell volume and apoptosis and has been shown to be downregulated in several types of cancer, including glioblastoma and melanoma. CaCC plays a role in cell migration and invasion and is overexpressed in pancreatic cancer. CLC has been shown to promote cell migration and invasion in breast cancer by regulating the activity of matrix metalloproteinases (MMPs).^[^ ^75^ ^]^

**Table 6:** Selected chloride channels and the implications of their altered expression in cancer.^[^ ^9^ ^]^

Cl^−^ channel type	Cl^−^ channel subtype	Inhibitors	Inhibition effect
Chloride‐leak (CLC)	CLC‐3	CLTX Anthraquinone emodin	Blocks cell shrinkage during invasion (glioma) Induces cell cycle arrest (nasopharyngeal carcinoma cells)
Chloride intracellular (CLIC)	N/A	N/A	Regulate cell motility, adhesion, apoptosis
Calcium‐dependent (CLCA)	N/A	N/A	Involved in pathophysiology of colorectal, pancreatic, and ovarian cancers

### 2.3 Faradaic Currents

Faradaic currents result from the electron transport generated in redox reactions happening across the cell membrane (Figure 5A). They are involved in cell‐cell communication mechanisms which contribute to maintain homeostasis in single cells and cell cohorts.^[^ ^76^ ^]^ Examples of electrochemical biomolecules involved in this interplay are NADH, NADPH, GSH, ascorbic acid, ubiquinone, and enzymes, due to their ability to accept or donate electrons.^[^ ^9^, ^77^ ^]^ Redox signaling is a key phenomenon involved in cell communication and observed both in plants and animals,^[^ ^78^ ^]^ with increasing evidence relating it to crucial life processes, such as ageing,^[^ ^79^ ^]^ cancer development,^[^ ^9^ ^]^ and immune response.^[^ ^80^ ^]^ Redox reactions are triggered by the presence of reactive oxygen species (ROS), which have a double nature: high ROS amounts can damage cellular components; lower ROS concentrations play a crucial role in establishing and regulating signaling pathways.^[^ ^78^ ^]^ It follows that the main molecules involved in signaling pathways by redox reactions are ROS‐generators.^[^ ^81^ ^]^ Most redox signaling occurs by the oxidation and reduction of reactive Cys residues. An example is the inactivation of the protein Tyr phosphatases.^[^ ^82^ ^]^ Intracellular sources include (i) mitochondria, the largest producers by ATP generation that culminates in the O_2_ reduction to water.^[^ ^83^ ^]^ (ii) NOX enzymes (the family of NADPH oxidases), where a flavin‐ and haem‐containing protein complex transfers electrons from cytosolic NADPH to molecular oxygen to produce superoxide anions.^[^ ^84^ ^]^ (iii) other enzymes, such as nitric oxide, xanthine oxidase, and cyclooxygenases, which produce ROS in lower amounts.^[^ ^85^ ^]^ (iv) other organelles. Examples are the endoplasmatic reticulum and the peroxisome. Other organelles are lower contributors, and their produced ROS amount varies based on the cell type.^[^ ^85^ ^]^

Cell redox maintenance plays a crucial role in cancer progression. For example, breast cancer cells exhibit higher faradaic currents when compared to their healthy counterparts.^[^ ^32^ ^]^ Similar phenomena have been observed in other types of cancer, including lung,^[^ ^63^ ^]^ pancreatic,^[^ ^86^ ^]^ and bladder^[^ ^87^ ^]^ cancer. By interfacing cancer cells with electrodes, it is possible to artificially control redox reactions.^[^ ^77^ ^]^ Bioelectrical manipulation strategies will be discussed in *Section* *8* of this review.

### 2.4 Cell Surface Charges

Surface charges can be defined as the net electricity observed in cells,^[^ ^88^ ^]^ and are given by the complex interplay of multiple factors, which are been subject of study for decades. Overall, researchers agree that the main contributors in the net electricity of a cell are i) charged molecules on the plasma membrane, such as glycolipids and glycoproteins, ii) ion channel activity, and iii) neutralizing systems in the body fluids, such as serum proteins.^[^ ^89^ ^]^ Cancer cells are characterized by a negatively charged surface profile due to their unique metabolic processes, known as the Warburg effect.^[^ ^90^ ^]^ Otto Warburg, a German biochemist, discovered in 1924 that healthy cells depend on the mitochondrial oxidative phosphorylation process to generate the required energy, while cancer cells rely on the glycolytic pathway that yields the secretion of lactic acid (Figure 5B).^[^ ^91^ ^]^ Cytoplasmic glycolysis and mitochondrial oxidative phosphorylation lead to the production of Adenosine triphosphate (ATP) in cells, used for storing and transmitting energy. According to the Pasteur effect, normal tissues produce the 90% of ATP by oxidative phosphorylation, and 10% by aerobic glycolysis.^[^ ^92^ ^]^ Cancer cells exploit the 80% of glucose to produce ATP solely via glycolysis, implying the intake of high levels of glucose to fulfil the energy requirements. In turn, this process produces large amounts of lactic acid (lactate), which facilitates the invasive ability of cancer cells and results in a negative net surface charge.^[^ ^93^ ^]^ In cancer cells, glutamine may also contribute to lactate production.^[^ ^94^ ^]^

Lactate in the human body is present in two stereoisomeric forms, D‐lactate and L‐lactate, with the latter being the most abundant.^[^ ^95^ ^]^ The physiological concentration range of lactate in healthy tissues is 1.5–3 mm,^[^ ^96^ ^]^ whereas in cancer tissues it reaches concentrations of 10–30 mm.^[^ ^97^ ^]^ This amount also depends on the glucose concentration in the microenvironment or in the culture medium. For example, breast cancer cells grown in high glucose media were found to produce high lactate concentrations, but when deprived of glucose they switched to consuming lactate for survival.^[^ ^98^ ^]^ Cervical cancer cells and human lung cancer cells use lactate as a carbon source to synthesize lipids.^[^ ^99^ ^]^ Nuclear magnetic resonance on lactate metabolism in vitro and in vivo revealed that it can be transported into cancer cells and being oxidized within them.^[^ ^100^ ^]^ Lactate itself cannot cross the plasma membrane by free diffusion, it requires a specific transport mechanism provided by monocarboxylate transporters (MCTs) which, besides lactate, can carry other monocarboxylates, such as pyruvate and ketone bodies.^[^ ^101^ ^]^ Over‐expression of lactate transporters was found to be a common feature of cancer cells.^[^ ^102^ ^]^ Overall, cancer cell metabolism yields the production of lactate anions that induce a negative net surface charge.^[^ ^12^ ^]^ This parameter is used as a biomarker in developing diagnostic methods, as discussed in *Section* *7* of this review.

## 3 Electrical Properties of Cancer Cell Populations and Tissues

The bioelectrical activity of single cells is propagated across cell cohorts by means of cell‐cell contacts, such as gap junctions. In this scenario, the electrical state of a cell affects the one of its neighbors which can be cancerous and non‐cancerous cells (see *Figure* *9*). Gap junctions are intercellular connections established by proteins called connexins, whose level of expression was found to vary among healthy and cancer cells.^[ ]^Findings on gap junctions in cancer are contradictory, suggesting that the mechanisms behind cell‐cell communications are driven by a variety of factors. For example, in the MDA‐MB‐435 breast cancer cell line, a lack of gap junctions has been correlated with increased metastasis and invasion,^[^ ^103^ ^]^ whereas gap junctions between brain carcinoma and astrocytes were shown to promote brain metastasis by cGAMP transfer.^[^ ^104^ ^]^ It follows that healthy and cancerous cells also exhibit dynamic communication among each other, and their signaling with tumor stromal cells gives them the ability to acquire pro‐tumoral phenotypes that can promote cancer progression. Unravelling the mechanism of interaction between cancer and the surrounding cells is key in cancer pathophysiology.^[^ ^25^ ^]^

**Figure 7:**
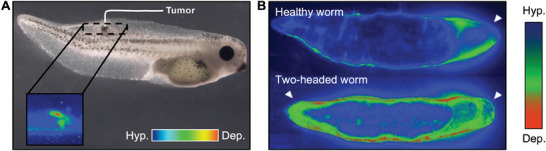
Bioelectrical effects at the single cell reflect to cell cohorts and whole tissues. A) Voltage‐sensitive dye imaging of cells abandoning their participation in organogenesis in favor of tumorigenesis, because of membrane depolarization. Reproduced under the terms of the CC‐BY licence.^[^ ^108^ ^]^ Copyrights 2013, the Authors. Published by The Company of Biologists. B) Bioelectrical pattern of resting potentials displays one depolarized region in wild‐type worms (healthy worm, top) and a mirror‐image bipolar pattern in worms that are or will be two‐headed (bottom). Reproduced with permission from.^[^ ^110^ ^]^ Copyrights 2021, the Authors. Published by the Royal Society.

In turn, cohort activities interfere with local ionic environments and transepithelial potentials, producing local electric fields across whole epithelia and tissues (**Figure** **7**).^[^ ^105^ ^]^ For example, significantly greater outward currents can be detected at the surface of tumor, compared to healthy tissues, and are responsible for the onset of migration and invasion dynamics.^[^ ^106^ ^]^ Interestingly, bioelectrical differences were also found between left and right sided breast cancer.^[^ ^107^ ^]^ Individual cells transforming into cancer depolarize as a very early step in the process. Figure 7A displays a voltage‐sensitive dyes imaging of depolarized cells decoupling from their mother tissue to undergo tumorigenesis.^[^ ^108^ ^]^ This is one of the explications of the broader concept of bioelectricity‐guided cellular behavior during morphogenesis.^[^ ^109^ ^]^ On the same line, investigations on bioelectricity at the embryonic stage introduced the new paradigm of bioelectric reprogramming. For example, it has been found that V_m_ prepatterns determine head number and position in planaria (Figure 7B).^[^ ^110^ ^]^ Transient perturbation via gap‐junction blockers that inhibit network connectivity or via ion‐channel drugs that redefine V_m_ values leads to a depolarized V_m_ pattern on both ends. This results in the growth of two‐headed worms. On the contrary, a converse change produces head‐less worms.^[^ ^111^ ^]^ From this perspective, cancer might be regarded as a physiological disorder caused by the failure of individual cells to join biological networks that work as multicellular systems toward common morphogenetic goals.^[^ ^109^ ^]^ Several technologies have been developed in the last decade to accurately detect and monitor the electrical activity in cancer cell populations and whole tissues, and are discussed in detail in *Section* *6* of this review.

## 4 Bioelectrical Phenomena Involved in Cancer Metastasis

Metastasis is the leading cause of death in patients with solid tumors, attributed to the migration of cancer cells to secondary organs in the body via the lymphatics, vasculature, or cerebrospinal fluid. Ion channels activity was found to be altered during the metastatic process, suggesting a clear correlation between the electrical activity of single cells, cell cohorts, and migration phenomena.^[^ ^112^ ^]^ The core events taking place in a metastatic cascade and driven by bioelectrical cues regard enhanced cell division, with consequent generation of cancer stem cells, and cell migration. These two phenomena are individually addressed in the following subsections.

### 4.1 Cancer Stem Cells

**Figure 8:**
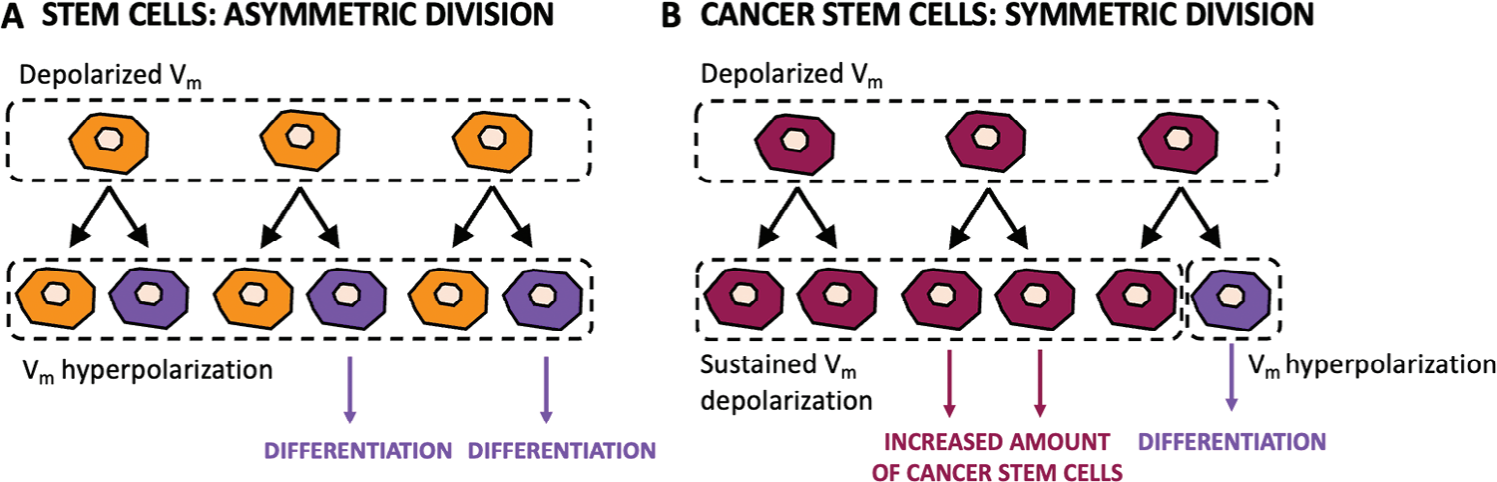
Comparison of the differentiation mechanism observed in healthy and cancerous stem cells. A) Healthy stem cells: asymmetric division, where each stem cell generates a copy identical to itself and another cell that will undergo differentiation and maturation. B) Cancer stem cells: symmetric division, where each stem cells produces two identical copies of itself, exponentially increasing the malignant cell reservoir. Redrawn from.^[^ ^14^ ^]^

It is widely confirmed that stem cells display unique electrophysiological patterns^[^ ^113^ ^]^ and express a variety of electrogenic transporters.^[^ ^114^ ^]^ Membrane hyperpolarization is thought to play a key role in their ability to differentiate.^[^ ^115^ ^]^ Conversely, direct cells electrical manipulation was shown to induce a stem‐like phenotype, suggesting that membrane depolarization may maintain cells in, or bring them back to, an undifferentiated stage at the gene expression level.^[^ ^116^ ^]^ Another study reports that misexpression in a wild‐type subunit of the KCNQ1 potassium channel has shown to confer neoplastic‐like properties to a stem cell subpopulation in the Xenopus embryo, by inducing over‐proliferation and migration.^[^ ^117^ ^]^ However, the functional significance of bioelectrical cues that guide stem cell behavior in complex morphogenetic events is largely mysterious.^[^ ^117^ ^]^ These biophysical signals are crucial aspects of the microenvironment that epigenetically regulate stem and tumor cell behavior. Cancer stem cells, a subpopulation of tumor‐like cells that can self‐renew and differentiate to form a tumor,^[^ ^37^ ^]^ are believed to play a leading role in tumor initiation, growth, and resistance to drugs and treatments.^[^ ^36^ ^]^ In some cases, they survive cancer therapies, making them the major cause of tumor recurrence.^[^ ^30^ ^]^ A major advantage for survival that cancer stem cells have compared to normal stem cells is given by their reproduction mechanism.^[^ ^118^, ^119^ ^]^ In contrast to healthy stem cells which reproduce by asymmetric division, where a stem cell produces one copy of itself and one cell that differentiates, cancer stem cells undergo symmetric division (**Figure** **8**).^[^ ^118^ ^]^ Here, each cancer stem cell generates two identical copies of itself, exponentially increasing the stem cell reservoir at each division. Some strategies targeting electricity of cancer stem cells have shown promising results. For example, by acting on the so called Wingless and Int‐1 (Wnt) pathways, a group of signal transduction exploiting cell surface receptors that regulate self‐renewal in cancer stem cells, was shown to decrease their chances of survival.^[^ ^37^ ^]^ Another approach is to target the metabolic pathways that are essential for their sustenance, such as the glucose metabolism.^[^ ^118^ ^]^

### 4.2 Cancer Cell Migration

**Figure 9:**
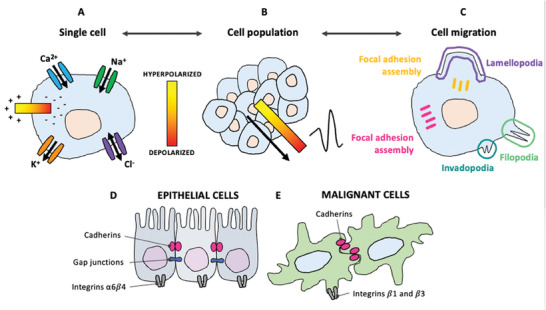
Cascade of bioelectrical events in cancer. A–C) An altered ion flux in single cancer cells with consequent V_m_ variation A) results in endogenous electric fields in cancer cell populations B), which in turn drive cells to migrate C). D,E) Morphological differences observed in epithelial healthy D) and malignant E) cells, responsible for the ability of cancer cells to migrate.

Cell migration under endogenous electric fields is known as galvanotaxis, and it drives phenomena such as wound healing and cancer metastasis (**Figure** **9**A–C).^[^ ^119^, ^120^ ^]^ The ability of cells to migrate under electrical gradients is a key process during the metastatic cascade.^[^ ^4^, ^121^ ^]^ Following the same principle, cell migration can be also artificially triggered by applying an external electric field to the epithelium. For example, it was demonstrated that an electric field of entity comparable to the trans‐epithelial potential (TEP) drives metastasis in multiple cancer cell lines.^[^ ^108^ ^]^ For instance, the MDA‐MB‐231 human breast cancer cell line undergoes electrotaxis via signaling through the EGFR in response to an applied electric field.^[^ ^122^ ^]^ In the case of cancer cells, the response to electric fields has been positively correlated to their metastatic potential.^[^ ^6^ ^]^ The core mechanism behind electrotaxis in healthy cells is an increase of intracellular calcium ions, which activates pathways associated to directional migration (e.g., receptor tyrosine kinases, PI3K, Rho GTPases, and ERK).^[^ ^119^ ^]^ Morphological variations are also key for cells to acquire the ability to migrate and survive in the bloodstream. Figure 9D,E illustrates morphological differences observed in healthy epithelial cells and malignant cells, highlighting the formation of cellular constructs that are necessary for cell migration. Sodium channels are also able to drive electrotaxis in human breast cancer cell lines, as upon the administering VGSC channel blockers (e.g., TTX), this phenomenon is reduced.^[^ ^30^ ^]^ In the same fashion, the K^+^ channel K_ir_4.2 responds to electric fields by facilitating PIP3 polarization to the leading edge of the migrating cell.^[^ ^123^ ^]^ Another key player in this scenario is the extracellular matrix (ECM). For example, glioblastoma cells migrate to the anode when cultured on poly‐L‐ornithine/laminin‐coated plastic, and to the cathode when embedded in a 3D hyaluronan/collagen hydrogel, suggesting that ECM composition influences the bioelectrical properties of the cell population.^[^ ^124^ ^]^ Electrical cues may also be used to detect cancer from distant sites of the body. This is attributed to the pre‐metastatic secretion of exosomes observed in cancer cells.^[^ ^125^ ^]^ Finally, in accordance with the enhanced ability of metastatic cells to migrate, studies report that a lower cell‐surface adhesion strength was positively associated to a higher metastatic potential in breast cancer cells.^[^ ^121^ ^]^

## 5 The Role of Nano‐Biotechnologies in Cancer Bioelectricity

This intermediate section aims at bridging what described so far in this review, i.e., cancer bioelectricity from a biological perspective, to the next sections, focused on nano‐biotechnologies applied to cancer electrophysiology, diagnostics, and therapeutics. At present, despite the numerous evidence on the altered electrical behavior of cancer cells, the scientific community lacks the comprehensive understanding of these electrical signals.^[^ ^109^ ^]^ For example, the bioelectrical signature of different types and subtypes of cancer cells compared to their healthy counterparts is still unknown. On the other hand, from an engineering perspective, the scientific community working in cellular electrical recording mainly targeted the development of cutting‐edge technological advancements to record action potentials in excitable cells.^[^ ^126^, ^127^, ^128^, ^129^, ^130^, ^131^ ^]^ This field of research is highly active and in constant expansion from the nanotechnology side. Microelectrode arrays record extracellular signals in excitable cells using highly precise plug‐and‐play systems operating at high resolutions. By customizing the electrodes and the readout systems, such devices would be able to record much tinier electrical signals in non‐excitable cells and open a whole new range of applications, as suggested by early experimental findings in the field.^[^ ^11^, ^29^, ^30^ ^]^ Besides fundamental investigations, bio‐nanotechnologies have been employed to harness what is known about cancer bioelectricity with the goal of detecting and manipulating these properties at the nanoscale in cancer diagnostics and therapeutics.^[^ ^9^, ^77^ ^]^ For example, controlling quantum signaling using bio‐nanoantennae has been shown to successfully induce cancer cell selective apoptosis.^[^ ^132^ ^]^ Nanoparticles were demonstrated to be a promising tool to identify and target cancer cells altered surface charges for diagnostic and therapeutic purposes.^[^ ^24^, ^133^, ^134^, ^135^ ^]^ To this regard, nanotechnology may have the potential of bridging the gap between biology and translational medicine. In particular, it could target at the same time scientific advancement and technological advancement as two independent, yet related, objectives:

- *Scientific Advancement: knowledge as the main goal*. Bespoke nanosystems may allow investigating cancer cells electricity in finer details and higher throughput both at the single cell and cell population level. The state‐of‐the‐art in the field of nanotechnologies for cellular electrophysiology has made substantial advancement in the last 30 years. Such nano‐platforms may be re‐designed to target non‐excitable cells, including cancer cells. In this case, the main application would be to broadening our fundamental knowledge. Examples are mapping cancer cell behavior, correlating electricity with cancer subtype, metastatic level, metastatic ability, and tumor localization.
- *Technological advancement: translational medicine as the main goal*. In parallel, based on the current knowledge about cancer cells bioelectricity and the progress made in the field of nanofabrication and nanosynthesis, such technologies can be exploited to develop nanosystems that can detect cancer cells electricity with high accuracy and specificity for diagnostic purposes, as well as reprogram bioelectricity at the nanoscale to develop novel strategies in cancer treatment. In this second case, the main application would be translational medicine.

The next sections present the latest advancement in nanotechnology for cellular electrophysiology (*Section* *6*), cancer diagnostics based on electricity (*Section* *7*), and cancer therapy based on electrical manipulation (*Section* *8*).

## 6 Nanotechnologies for Cellular Electrophysiology

Non‐excitable cells exhibit static electric signals, orders of magnitude smaller than action potentials observed in excitable cells. This makes it more challenging to perform electrophysiological recording of non‐excitable cells using existing technologies. The gold standard to measure bioelectric properties of individual cells are patch clamp, microelectrode arrays, impedance spectroscopy, and fluorescence imaging.^[^ ^136^ ^]^ Patch clamp accurately measures ion channel activity and membrane potential changes in individual cells, and its inventors were awarded the Nobel Prize in 1991.^[^ ^137^ ^]^ This high‐sensitivity method involves placing a glass micropipette on the cell membrane to record intracellular signals. However, it provides low throughput. Microelectrode arrays (MEAs), in turn, allow to achieve high throughput by simultaneously recording the extracellular electrical activity of multiple cells with tens of electrodes, and are regarded to as a low‐sensitivity technique compared to patch clamp. Nevertheless, MEAs are designed to record higher intensity (and frequency) time‐resolved signals in excitable cells, and this technology at present has not been translated to record static signals in non‐excitable cells.^[^ ^138^ ^]^ Impedance spectroscopy involves measuring the electrical impedance of cells in response to an applied electrical current. By analyzing the frequency‐dependent impedance of individual cells, it is possible to extract information about cell size, shape, and electrical properties.^[^ ^139^ ^]^ Fluorescence imaging involves the use of fluorescent markers, such as voltage‐sensitive dyes, to optically image membrane potential changes. Overall, all these methods present some limitations. Patch‐clamp requires physical access to the cytoplasm, it is a low‐throughout method, and it is not designed to perform long‐term continuous recordings. Impedance spectroscopy requires the application of a stimuli, which, in the case of signals of very small entity, may induce cell behavioral changes and lead to falsified results. Finally, fluorescent dyes rely on a direct chemical interaction with the cell, which is undesirable for monitoring the natural state and activity of the same. In addition, most of these methods do not consider the cumulative bioelectrical activities observed in cancer cell cohorts and whole tissues.^[^ ^11^, ^29^, ^30^ ^]^ To address these bottlenecks, novel technologies are being developed, and they are individually addressed in the next subsections.

### 6.1 Large Area Electrodes

**Figure 10:**
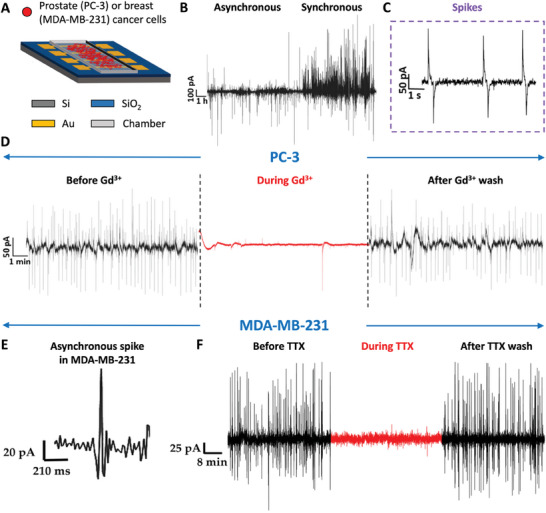
Large‐area, low‐impedance electrodes to record the low‐frequency electrical activity of cancer cell cohorts in cancer cell lines. A) Schematic of the microelectrodes. Reproduced under the terms of the CC‐BY licence.^[^ ^29^ ^]^ Copyrights 2019, the Authors. Published by MDPI. B) Most representative electrical activity of PC‐3 cells, showing a combination of asynchronous and synchronous activity. Reproduced under the terms of the CC‐BY licence.^[^ ^29^ ^]^ Copyrights 2019, the Authors. Published by MDPI. C) Current spikes recorded in the prostate cancer cell line PC‐3. Reproduced under the terms of the CC‐BY licence.^[^ ^29^ ^]^ Copyrights 2019, the Authors. Published by MDPI. D) 20 min continuous recording of the prostate cancer cell line PC‐3, before and after administering sodium channel blocker tetrodotoxin (TTX). Reproduced under the terms of the CC‐BY licence.^[^ ^29^ ^]^ Copyrights 2019, the Authors. Published by MDPI. E) Asynchronous spike recorded from the highly metastatic breast cancer cell line MDA‐MB‐231. Reproduced under the terms of the CC‐BY licence.^[^ ^30^ ^]^ Copyrights 2020, the Authors. Published by Frontiers. F) Voltage‐gated sodium channel activity in MDA‐MB‐231 breast cancer cell line recorded via current measurement across a cell cohort, before and after administering sodium channel blocker tetrodotoxin (TTX). Reproduced under the terms of the CC‐BY licence.^[^ ^30^ ^]^ Copyrights 2020, the Authors. Published by Frontiers.

Large area electrodes come into play with the aim of monitoring unbiased cell activity at a population level, which is critical to understand cohort effects that drive tumor proliferation. Both single cell and cell populations in cancer exhibit lower currents (in the range from 0.1 to 100 pA) and at lower frequencies (1–10 Hz) compared to excitable cells. This implies that a main barrier to the investigation of these phenomena derives from the technological challenge associated to it.^[^ ^30^ ^]^ Therefore, commercially designed micro (multi) electrode arrays employed in neuronal recording cannot be translated to be operated with non‐excitable cells. One approach to address the issue revolves around measuring the current flowing between two electrodes with larger area, which results from the voltage drop observed in presence of a cell cohort.^[^ ^11^, ^30^ ^]^ This approach was found to enable targeting signals at lower frequencies and with higher precision, given by the lower impedance of the electrodes (**Figure** **10**). The measurable current *i_S_* (*t*) is defined as:

(2) $$i_{s}\left(t\right)=\frac{dv_{s}}{dt}\cdot C_{D}\left(1-e^{-\frac{t}{\tau }}\right)$$

where τ is the time constant, and the capacitance C_D_ acts as a multiplying factor that, being it directly proportional to the electrode area, in this case it allows for the amplification of the signal. Changes on the extracellular potentials (*v_s_*) results in variations in the measurable current.^[^ ^30^ ^]^ Equally spaced gold round electrodes, each connected to its measuring pad, were obtained on a Silicon/Silicon oxide wafer by shadow mask lithography (Figure 10A).^[^ ^11^, ^29^, ^30^ ^]^ This method was implemented in prostate and breast cancer cell lines. Prostate cancer PC‐3 cell cohorts exhibited a cumulative electrical fingerprint given by asynchronous spikes followed by a synchronized spiking pattern (Figure 10B). Typically observed synchronous spikes are shown in Figure 10C. By performing long‐term recording (20 min), the authors showed that the electrical activity of the prostate cancer cell line PC‐3 was suppressed by administering the sodium channel blocker tetrodotoxin (TTX), suggesting that the activity revolves around sodium channels. The same approach was implemented in breast cancer cell lines MDA‐MB‐231 and MCF‐7, highly and poorly metastatic, respectively. It was found that the intensity and frequency of the electrical activities, measured in the form of current spikes recorded from cell populations across microelectrodes, were correlated to the metastatic potential. MCF‐7 cells exhibited poor to absent current spiking,^[^ ^30^ ^]^ as opposed to MDA‐MB‐231 highly metastatic cells. Figure 10E presents a typical spike recorded from MDA‐MB‐231 cell line. Figure 10F displays that the electrical activity is attributed to voltage‐gated sodium channels, as confirmed by current recordings before and after the administration of tetrodotoxin.^[^ ^30^ ^]^ Hence, the system could discriminate the metastatic ability of the examined cells, associated to a recurring current spiking.^[^ ^30^ ^]^

### 6.2 3D Nanoelectrodes

**Figure 11:**
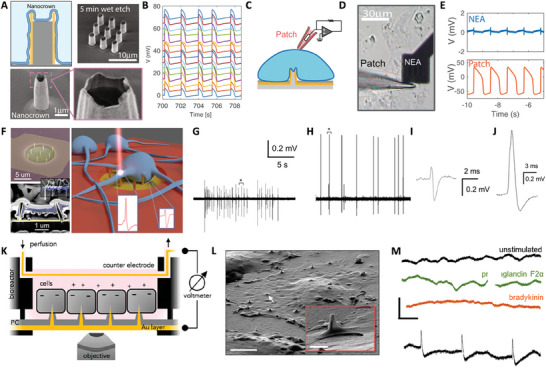
3D nanoelectrodes in electrophysiological recording. A–E) Nanocrown electrodes for recording action potentials in cardiomyocytes. Reproduced under the terms of the CC‐BY licence.^[^ ^144^ ^]^ Copyrights 2022, the Authors. Published by NPG. A) Schematics and SEM micrographs of nanocrown electrodes. B) Sample parallel recording from nanocrown electrodes. C) Sketch of the conditions for simultaneous patch‐clamp and nanocrown electrodes recording. D) Microscope image of cardiac cells on a nanoelectrode during patch‐clamp recording. E) Action potentials simultaneously recorded with nanoelectrode arrays and patch‐clamp, demonstrating the functionality of the technology. F–J) 3D nanoelectrodes combined with flat microelectrodes and laser poration to record intra and extracellular activity in mammalian neurons. Reproduced under the terms of the CC‐BY licence.^[^ ^152^ ^]^ Copyrights 2017, the Authors. Published by the American Chemical Society. F) SEM micrographs of nanopillars on microelectrodes (top left) and neuron engulfing nanopillars (bottom left). At the right, a sketch of the working principle is presented. G) Typical extracellular recording from microelectrodes. H) Typical intracellular recording following laser poration. I) Individual extracellular spike taken from G. J) Individual intracellular spike taken from H. K–M) Nanopillars on large‐area electrodes for simultaneously monitoring the intracellular activity of NRK cell populations (non‐excitable). Reproduced under the terms of the CC‐BY licence.^[^ ^150^ ^]^ Copyrights 2019, the Authors. Published by the American Chemical Society. K) Device schematic and working principle. L) SEM imaging of NRK cells on nanopillars. Scale bar: 5 µm; inset scale bar: 1 µm. M) Voltage recordings under pharmacological stimulation after confluency, to target calcium action potentials: 1 µm PF2 (green), 1 µm BK (orange), unstimulated (black). Scale bars: 0.6 mV, 3 min. At the bottom, voltage recordings of NRK cells (black) and plane gold control (red). Scale bars: 0.5 mV, 60 s.

3D electrode geometries have been coupled to microelectrode arrays and employed in the last decade to establish a tight cell‐electrode contact.^[^ ^140^ ^]^ On one hand, this method allows for obtaining extracellular recordings with higher precision. At the same time, by designing the electrode tip according to the application of interest, the intracellular compartments may be accessed with a minimally invasive approach. Vertical nanostructures, such as sharp pyramids and nanopillars with high aspect ratio fabricated over commercial microelectrode arrays, were previously demonstrated to record action potentials in excitable cells at the single‐cell level with remarkable accuracy.^[^ ^126^ ^]^ These approaches revolutionized the field of electrophysiological recording, and have been reviewed elsewhere.^[^ ^126^, ^130^, ^131^, ^138^, ^141^, ^142^, ^143^, ^144^, ^145^, ^146^, ^147^, ^148^, ^149^ ^]^ To this regard, two important works are highlighted in this review for brevity, namely the use of nanocrown electrodes to record action potentials in cardiomyocytes (**Figure** **11**A–E), and 3D plasmonic nanoelectrodes integrated with MEAs to record action potentials in mammalian neurons and cardiac cells (Figure 11F–J). The application of nanoelectrodes to the recording of non‐excitable cells population is further presented (Figure 11K–M).^[^ ^150^ ^]^ Nanoelectrodes were also integrated with CMOs MEAs and microfluidics to record the activity of thousands of interconnected neurons.^[^ ^130^ ^]^ Other works in the field include the use of porous microelectrode materials combined with ultrafast laser excitation to record extracellular and intracellular signals in single excitable cells.^[^ ^127^, ^138^, ^151^ ^]^

Nanocrown electrodes were obtained via cutting‐edge photolithography methods to yield heterostructures of platinum (bright) and SiO_2_ (dark) (Figure 11A).^[^ ^144^ ^]^ Arrays of nanocrown electrodes allowed to record electrophysiological signals from tens of cardiomyocytes simultaneously with high accuracy (Figure 11B). To validate the functionality of the system, patch‐clamp recordings were performed concomitantly with electrical recordings on nanoelectrodes in the configuration depicted in Figure 11C, and Figure 11D displays a micrograph of the said measurement. As further shown in Figure 11E, the measurement confirmed the reliability of the proposed nanocrown electrodes as efficient nanosystems for continuous electrophysiological recording.

To achieve unperturbed long‐term recording of intra‐ and extracellular activity in single neurons with high signal‐to‐noise ratio, the combination of nanostructures and laser poration on microelectrodes was demonstrated to be a successful approach.^[^ ^152^ ^]^ Nanoelectrodes promote intimate contact between the recording area and the cells, and laser poration acts as a minimally‐invasive method to induce pillar insertion into the contacting cell, while preserving the sealing. In this scenario, the underneath microelectrode interfaces with the extracellular environment, and the nanopillar contacts the intracellular compartment, allowing for the simultaneous recording of intra‐ and extracellular activity. Figure 11F presents micrograph of the nanopillars, both bare and in contact with neurons, and a sketch of the working mechanism of the nanosystem. An example of recorded electrical activity is displayed in Figure 11G (extracellular) and Figure 11H (intracellular, following laser poration). Individual spikes are zoomed and presented in Figure 11I and Figure 11J, respectively.

The methods presented above may be translated to the long‐term recording of cancer cells electrical activity, and have recently inspired further applications of 3D nanostructures in non‐excitable cells and cell cohorts (Figure 11K–M).^[^ ^7^, ^150^ ^]^ For example, nanopillars were deposited on large‐area electrodes and used to simultaneously access the cytoplasm of multiple non‐electrogenic cells. The device schematic and working principle is illustrated in Figure 11K. Figure 11L presents a SEM micrograph of NRK cells on nanopillars, where the capacity of such nanostructures to establish a tight contact with cells is visible. The electrophysiological activity of NRK cells on nanopillars was obtained by voltage recordings without medium perfusion. After confluency, the source of the electrical activity was evaluated by pharmacological administration targeting calcium channels, using prostaglandin F2α and bradykinin (Figure 11M). Overall, 3D nanoelectrodes are now well established in the recording of action potentials in excitable cells. By decreasing electrode impedance to minimize the noise contribution at low frequencies, steps are being made toward the translation of this technology to the recording of the electrical activity of non‐excitable cells.^[^ ^7^ ^]^

### 6.3 Optical Nanotechnologies in Cellular Electrophysiology

Compared to the fast voltage changes observed in neurons and muscle cells, non‐excitable cells exhibit slow and low‐intensity voltage changes, which are less likely to be detectable.^[^ ^11^ ^]^ It follows that the main requirements to fulfil in non‐excitable cells recording technologies are i) high sensitivity of the probe, ii) long‐term recording capabilities, and iii) minimized invasiveness. Optical technologies have been suggested as a promising technique to address such challenges, and are based on the use of voltage‐sensitive indicators, which comprise voltage‐sensitive dyes and genetically encoded voltage indicators.^[^ ^153^ ^]^ Other optical technologies revolve around the combination of 3D electrodes with dyes, and electrochromic electrode materials, both providing an optical readout of cellular electrical signals.^[^ ^7^, ^129^ ^]^

#### 6.3.1 Voltage‐Sensitive Indicators

Voltage‐sensitive indicators can be classified in two subcategories: voltage‐sensitive dyes and genetically encoded voltage indicators.^[^ ^153^ ^]^ The first act by establishing chemical bonds with molecules expressed by cell membranes to generate a voltage‐dependent optical signal,^[^ ^153^ ^]^ and have been deployed for measuring fluctuations in non‐excitable cells as well as to follow voltage dynamics across single neurons or cortical areas.^[^ ^153^, ^154^ ^]^ Genetically encoded voltage indicators (GEVIs) were further developed to allow targeting of cell type‐specific promoters in genetically tractable cells or organisms.^[^ ^154^, ^155^ ^]^ Hybrid voltage indicators, which combine a voltage‐sensitive dye with a genetically encoded component, have been also developed and are discussed elsewhere.^[^ ^156^ ^]^

##### Voltage‐Sensitive Dyes

**Figure 12:**
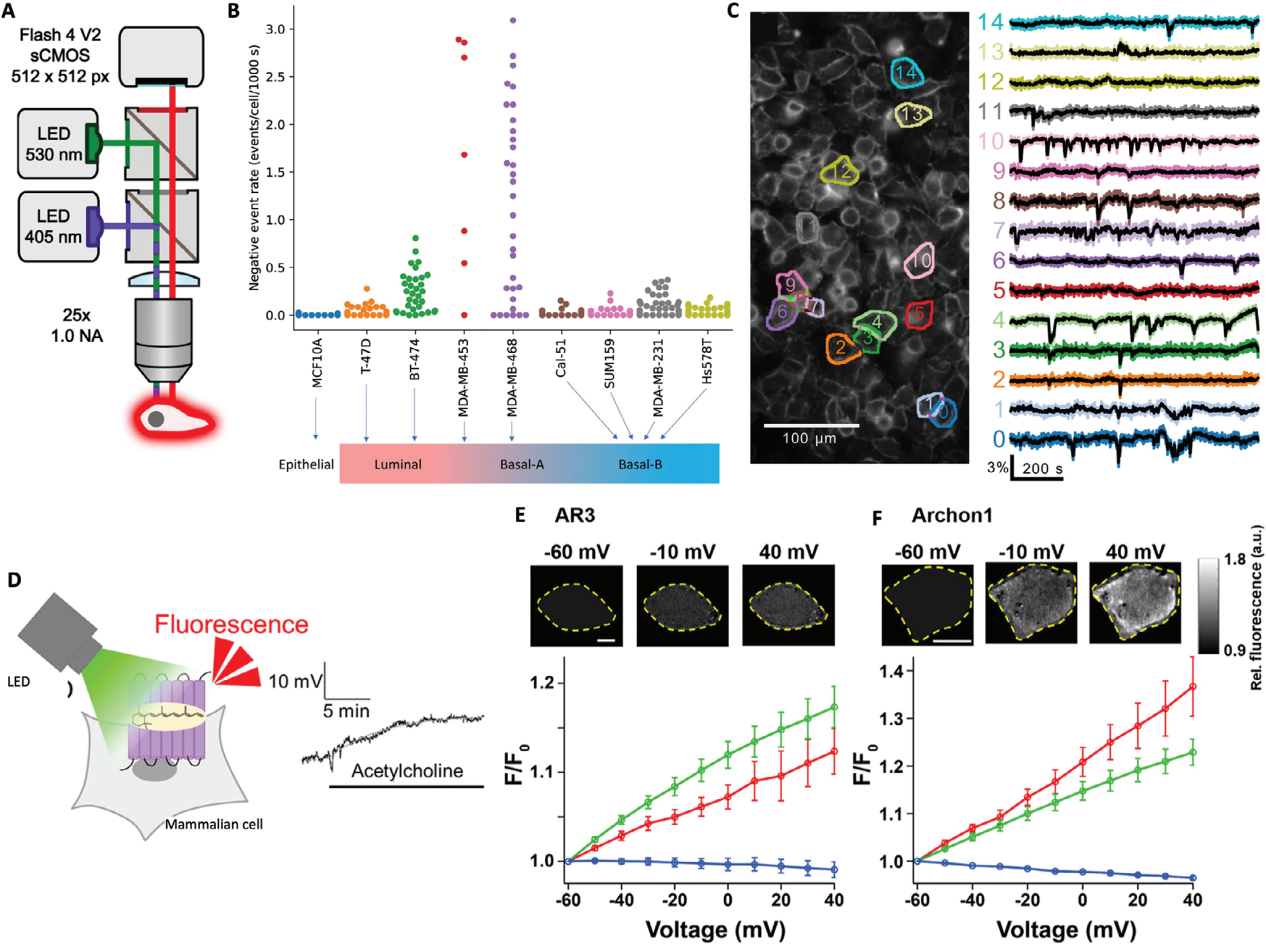
Voltage‐sensitive indicators for non‐excitable cells electrophysiological recording. A–C) Voltage imaging of single breast cancer cells. Reproduced under the terms of the CC‐BY licence.^[^ ^15^ ^]^ Copyrights 2022, the Authors. Published by NPG. A) Schematic of the widefield epifluorescence imaging system with two‐color excitation. B) Mean spiking events for each cell line in 1000s in the adopted field of view. C) Voltage fluctuations imaged in MDA‐MB‐231 single cells during 15 min recordings. D–F) Voltage‐sensitive fluorescence of AR3 and its high fluorescence mutant Archon1 in mammalian cell lines using low‐intensity LED stimulation. Reproduced under the terms of the CC‐BY licence.^[^ ^157^ ^]^ Copyrights 2023, the Authors. Published by the American Chemical Society. D) Schematics of the setup and working principle. E) Voltage‐dependent changes in fluorescence of AR3 in mammalian cells. F) Voltage‐dependent changes in fluorescence of Archon1 in mammalian cells. Cells are outlined in yellow and the outside in black. Scale bars: 10 µm.

A recent study reports on the use of voltage‐sensitive dye di‐4‐AN(F)EP(F)PTEA, which binds the outer cell membrane and shifts its absorption and emission spectra as a function of the potential with sub‐microsecond resolution (**Figure** **12**A). Such dye was administered to breast cancer cell lines to dynamically light up the electrical activity of the different subtypes subtypes in hundreds of cells simultaneously. Signal amplification was achieved by sequentially exciting the dye with blue and green LEDs, which allowed to reach high throughput, dynamic optical recording. The authors report that Luminal B and Basal A cell lines confirmed31 to be the most active, in contrast with the healthy control MCF10A (Figure 12B). Among the findings, spontaneous V_m_ fluctuations recorded at 5 frames per s in cultured MDA‐MB‐231 monolayers displayed that most cells exhibited few or no fluctuations, but a subset of cells was highly active (Figure 12C). Such activity could be reduced by administering sodium channel blockers or calcium‐activated potassium channel blockers. Voltage‐sensitive dyes are a convenient approach that allow long‐term and high‐sensitivity recording of cell membrane potentials. However, they may alter cellular behavior due to establishing a chemical bond with cells, and occasionally induce toxic effects.^[^ ^137^ ^]^

##### Genetically Encoded Voltage Indicators

Genetically encoded voltage indicators (GEVIs) may be a promising alternative in this direction, providing high specificity.^[^ ^153^, ^157^ ^]^ GEVIs have been extensively employed to record the electrical activity of the brain with enhanced sensitivity and temporal resolution compared to calcium indicators, and this field of research is currently active toward the development of new indicators.^[^ ^128^, ^154^, ^158^, ^159^ ^]^ The main requirements for a GEVI to record electrical fluctuations in cells are photostability, brightness, speed of operation, sensitivity, and specificity, to achieve one and two‐photon voltage imaging.^[^ ^160^ ^]^ Recently, a low‐intensity LED was used as the light source to detect slow changes in voltage using rhodopsin‐based genetically encoded voltage indicators (GEVIs) (Figure 12D–F).^[^ ^148^, ^155^ ^]^ The working principle is presented in Figure 12D. Two indicators were employed, namely AR3 and Archon1, and allowed for the optical detection of mammalian cell potentials as presented in Figure 12E and Figure 12F, respectively. This method allowed to successfully detect lower voltage‐sensitive fluorescence intensity in different mammalian cell lines for 20 min. These preliminary findings suggest that combining rhodopsin‐based GEVIs with low‐intensity LED stimulation is a potential approach worth further investigation for the long‐term voltage imaging of non‐excitable cells.^[^ ^157^ ^]^ Other examples of recently developed GEVIs are JEDI‐2P, which reported light‐evoked responses in Drosophila interneurons and in the mice retinas, as well as at the single‐neuron and neuronal pair level,^[^ ^161^ ^]^ and protein indicators for high‐throughput cardiomyocytes recording,^[^ ^162^ ^]^ which are extensively reviewed elsewhere.^[^ ^128^, ^154^ ^]^ Video‐based screening of pooled libraries can be a powerful approach to target photoselection over a large field of view, enhancing the capabilities of imaging technologies.^[^ ^163^ ^]^ A recent example involved the use of archaerhodopsin‐derived GEVIs to obtain live voltage imaging of mouse brains with improved signal‐to‐noise ratio (QuasAr6a) and kinetics (QuasAr6b). Such method may be translated to non‐excitable cells and tissues. Optogenetic methods for voltage recording are showing to be a promising avenue in the field of non‐excitable cells electrophysiology.^[^ ^109^, ^154^ ^]^

#### 6.3.2 Combined Approaches

**Figure 13:**
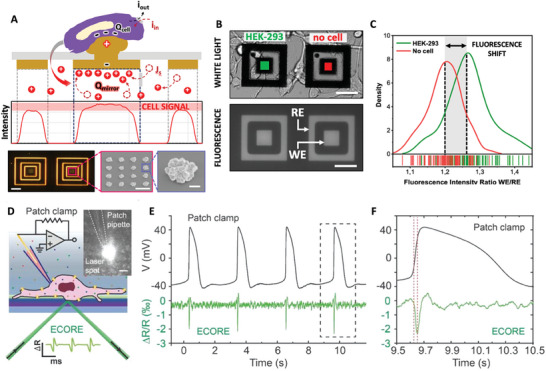
Combined approaches: electrodes with optical readout. A–C) Passive recording of bioelectrical signals in HEK‐293 cells by fluorescent mirroring. Reproduced under the terms of the CC‐BY licence.^[^ ^7^ ^]^ Copyrights 2023, the Authors, Published by the American Chemical Society. A) Working principle of the technology: the interfacial charge of the electrical double layer established at the gold‐electrolyte interface mirrors the electrical activity of the cell sitting on top of the 3D nanoelectrode on the top chamber of the device. The insets show an image of a pair of electrodes in dark field as seen from the bottom chamber. The dots at the central electrode correspond to the 3D electrodes observed on the other end of the membrane. Scale bars (from left to right): 30 µm, 2 µm, 300 nm. B) Images of the electrode pairs taken with an optical microscope (white light) versus a confocal microscope (fluorescence). C) Distribution plot of the fluorescence emitted by bare electrodes compared to electrodes in contact with a HEK‐293 cell. D–F) Recording the electrical activity of cardiomyocytes monolayers via electrochromic materials. Reproduced under the terms of the CC‐BY licence.^[^ ^129^ ^]^ Copyrights 2022, the Authors. Published by the American Chemical Society. D) Schematic of the simultaneous recording with electrochromic PEDOT:PSS thin films and patch clamp from the same cell. Inset scale bar = 50 µm. E) Synchronized intracellular (patch clamp) and extracellular (PEDOT:PSS) recordings. F) Zoom at a single intra and extra cellular signals, displaying extracellular spikes during cell membrane depolarization.

Recent advancements demonstrated the combination of nanoelectrodes with dyes or electrochromic electrode materials to obtain optical readouts of cellular signals. An example is shown in **Figure** **13**A–C, where the adopted approach brings together the advantages of exploiting 3D nanoelectrodes and fluorescent charged molecules, while overcoming the limitations of both.^[^ ^7^ ^]^ Nanoelectrodes were used as the vehicle to transport the cell electrical activity in a passive configuration, and dyes were decoupled from the biological medium to avoid direct interaction with cells. This is achieved by implementing pass‐through conductive nanostructures across a suspended membrane, which defines two different chambers. At the top, cells are cultivated over 3D nanoelectrodes. The cell polarizes the 3D electrode head down to the planar electrode tail at the bottom chamber, which in turn triggers the migration of charged fluorophores at the interface, yielding to fluorescent emission of intensity that mirrors the cell electrical contribution. The static charge at the electrical double layer observed at the interface planar electrode‐fluorescent dispersion is proportional to the cell signal. By implementing surrounding planar electrodes at the bottom chamber only, the optical noise can be filtered out (Figure 13A). The inset show a micrograph of an electrode pair as seen from the bottom chamber, where working electrode (central, pass‐through) and reference electrode (surrounding, bottom chamber only) are visible. The dots observed in dark field in the central electrode area are the 3D nanoelectrode heads at the top chamber, as shown in the zoomed insets. Figure 13B presents the same electrode pair as seen from the bottom chamber, after cultivating cells on the top chamber. The top image was taken under white light illumination, and the bottom image under fluorescence excitation. The ratio between the central electrode and its surrounding square is a measure of the cell signal, which was found to be altered of the ≈6% in electrodes in contact with HEK‐293 cells when compared to bare electrodes (Figure 13C).

Another promising avenue in the field of optical recording of cellular electrical signals is the use of electrochromic electrode materials, that exhibit a colorimetric behavior in response to an applied mechanical force. PEDOT‐PSS thin films were recently synthetized and interfaced with cardiac cells to record action potentials resulting from the spontaneous and rhythmic contraction of cardiomyocytes monolayers.^[^ ^129^ ^]^ A dual‐color approach was adopted to probe single cells using a 25 µm probing beam. Figure 13D shows a schematic of the simultaneous intracellular recording using patch‐clamp and extracellular using electrochromic PEDOT films from a cardiac cell, with the inset displaying the concurrent probing beam and patch needle on the same cell. The resulting sample recording is presented in Figure 13E, where the synchronized intra and extra cellular signals are plotted. A zoom on a single event is reported in Figure 13F, where it is possible to notice the optically recorded extracellular spike in synchrony with membrane depolarization recorded by patch clamp.

## 7 Cancer Diagnostics Based on Electricity

Traditional cancer detection technologies often involve multiple steps such as sample treatment, molecular amplification, and target detection. In the last decades, multiple technologies have been developed to facilitate these processes and reduce their costs.^[^ ^164^ ^]^ For example, nanotechnologies were employed to aid the diagnosis of oral cancer,^[^ ^135^ ^]^ breast cancer,^[^ ^165^ ^]^ prostate cancer,^[^ ^165^ ^]^ and ovarian cancer.^[^ ^166^ ^]^ There is a vast literature on cancer diagnostics based on fluorescence,^[^ ^87^, ^167^, ^168^ ^]^ colorimetry,^[^ ^133^ ^]^ chemoluminescence,^[^ ^169^ ^]^ surface enhanced Raman scattering (SERS),^[^ ^170^ ^]^ surface charge sensing,^[^ ^91^ ^]^ and electrochemical sensing,^[^ ^165^, ^171^ ^]^ to detect nucleic‐acids,^[^ ^172^ ^]^ aptamers,^[^ ^173^, ^174^ ^]^ and other cancer biomarkers.^[^ ^175^ ^]^ These involve the employment of different materials and devices, including nanoparticles,^[^ ^135^, ^174^, ^176^, ^177^ ^]^ field‐effect transistors,^[^ ^164^ ^]^ and 2D materials.^[^ ^178^, ^179^, ^180^ ^]^ In this review, selected recent advancements are presented based on their action mechanisms which involve cancer bioelectricity. The main players in this scenario are novel methods for surface charge sensing, i.e., detecting the negative charge present on cancer cells membranes. As discussed in *Section* *2.3*., cancer cells undergo the Warburg effect, which results in the production of lactate anions and the establishment of a negative surface charge on the cell membrane. The detection of this charge has been recently targeted to isolate cancerous cells from healthy cells. However, cell surface properties are not unique to cancer cells, and this is the main limitation imposed by this approach.^[^ ^91^ ^]^ Charged nanoparticles are one of the main technologies under development to selectively bind cancer cells in tissue samples or in the bloodstream.^[^ ^89^, ^181^ ^]^ They can be designed according to the specific application, mainly for targeting, imaging, and sensing purposes.^[^ ^24^, ^133^, ^134^, ^174^, ^182^ ^]^ (i) *Targeting*. By functionalizing the nanoparticles with a suitable chemistry, such as antibodies, cancer cells can be bound with high specificity and sensitivity.^[^ ^133^, ^174^ ^]^ (ii) *Imaging*. The nanoparticle‐cell complex is designed such to exhibit optical properties which allow its selective visualization using imaging techniques, including magnetic resonance imaging (MRI) or computed tomography (CT) scanning.^[^ ^87^, ^164^, ^183^ ^]^ (iii) *Sensing*. Charged nanoparticles can also be used to detect cancer biomarkers. For example, some nanoparticles are designed to bind to circulating tumor cells (CTCs) in the bloodstream to monitor cancer progression. Additionally, charged nanoparticles can be engineered to carry drugs or other therapeutic agents directly to cancer cells, which can improve the efficacy of cancer treatments and reduce side effects.^[^ ^135^, ^168^, ^170^ ^]^

**Figure 14:**
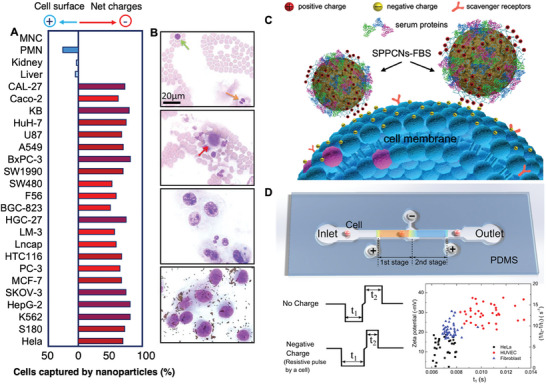
Cancer screening based on surface charges. A) Net captured percentage of cells by one type of nanoparticle (positive) subtracted by the cells captured by the nanoparticle opposite charge sign (negative), used as controls. Reproduced under the terms of the CC‐BY licence.^[^ ^89^ ^]^ Copyrights 2016, the Authors. Published by Ivy Springs. B) Microscope photographs of blood smear with cancer cells. From top to bottom: MNC cell, green; PMN cell, orange; MDA‐MB‐231 cells, red; MDA‐MB‐231 cells smear; positive NPs captured MDA‐MB‐231 cells from blood. Reproduced under the terms of the CC‐BY licence.^[^ ^89^ ^]^ Copyrights 2016, the Authors. Published by Ivy Springs. C) Schematic illustration of the binding of cancer cells by the protein−SPPCNs complexes. After adsorption of the serum proteins, parts of the positively charged surface of the SPPCNs are still exposed and available for interacting with the negatively charged cancer cell membrane. Reproduced under the terms of the CC‐BY licence.^[^ ^181^ ^]^ Copyrights 2020, the Authors. Published by the American Chemical Society. D) Schematic of the microfluidic sensor for in situ cell surface charge measurement. (i) Illustration of the two‐stage resistive pulse sensing structures for cell surface charge measurement. (ii) Illustration of a typical resistive pulse signal when a cell passes the 2‐stage RPS consisting of a negative pulse and a positive pulse, separated by an interval with alleviated slope. (iii) (1/t2‐1/t1) and corresponded zeta potential versus t1 for different cells. The star represents statically significant difference between two groups with p less than 0.05. Reproduced under the terms of the CC‐BY licence.^[^ ^90^ ^]^ Copyrights 2020, the Authors. Published by the American Chemical Society.

Fluorescent superparamagnetic nanoprobes were synthetized and employed to target 22 different cancerous and 4 healthy cell lines without the aid of molecular biomarkers, with the aim of discriminating the cell type based on its surface charge resulting from glucose metabolism. An elevated glycolysis in the cancer cell was correlated with an increased lactate secretion. Nanoprobes bonded with all cancer cells and did not attach to any of the healthy cells (**Figure** **14**A).^[^ ^89^ ^]^ Human breast cancer cells (MDA‐MB‐231) were also mixed with whole blood to confirm the functionality of the system. Figure 14B shows the typical field of normal blood smear, the normal blood spiked with MDA‐MB‐231 cells, MDA‐MB‐231 cells, and MDA‐MB‐231 cells recovered by the positive nanoprobes. This study demonstrated an efficiency of cancer cell recovery above the 90%. Similarly, super‐paramagnetic fluorescent nanoparticles have been employed to selectively bind CTCs based on their surface charge (Figure 14C). Upon cancer cells−nanoparticle interaction via optimum incubation, serum protein‐coated nanoparticles could trap different cancer cells in blood independent of their epithelial protein expression.^[^ ^181^ ^]^ Identification techniques include immunostaining fluorescence, in situ hybridization, and immunofluorescence in colorectal cancer patients and healthy volunteers. Nanoprobes have also emerged in recent years to study the cellular surface properties with increased sensitivities.^[^ ^89^ ^]^

Microfluidic technology has also been adopted to sense surface charges and sizes of single cancer cells in continuous flow. Opposite electric fields were applied to two consecutive resistive pulse sensors. Electric fields determined an acceleration or deceleration of charged cells making it possible to differentiate them according to the transit time. The method was tested on HeLa cancer cells, human dermal fibroblast cells, and human umbilical vein endothelial cells (HUVECs) (Figure 14D).^[^ ^90^ ^]^ Overall, the development of non‐invasive technologies that can selectively identify cancer cells within tissues and the bloodstream hold great promise as an alternative to current methods in cancer diagnostics.

## 8 Cancer Therapies Based on Electrical Manipulation

The alterations in bioelectric properties observed in cancer cells make them an attractive target for cancer therapy. Several strategies are being explored for targeting ion channels and other signaling pathways. For example, small molecule inhibitors have been developed to selectively block processes that contribute to cancer cell growth and survival. Other studies have investigated the use of electrical stimulation to alter the bioelectric properties of cancer cells and promote apoptosis. Cellular bioelectricity has also been shown to represent a promising target in tissue engineering and regeneration.^[^ ^184^ ^]^ Therapeutic approaches that are discussed hereby include therapeutic nanoparticles, tumor treating fields (TTFs), and electroceuticals.

### 8.1 Therapeutic Nanoparticles

Current cancer immunotherapies have demonstrated significant clinical success, but always suffer from immunotoxicity and autoimmune disease. Recently, nanomaterial‐based immunotherapies have been developed to precisely control in vivo immune activation in cancerous tissues. The mechanism of interaction between immune cells and cancer cells remains unclear. In this direction, nanoparticles have been employed as immuno‐modulating agents, to modify the cancer cell surface using natural killer cell (NK cell)‐activating signals.^[^ ^182^ ^]^ In a recent study, inhibition of tumor growth was observed in mice without noticeable side effects.^[^ ^24^ ^]^ Viral vaccine‐loaded nanoparticles were also shown to delay tumor progression and prolong survival in a HER2+ tumor mouse model.^[^ ^185^ ^]^ Recently, various nanomaterials with unique optical and electrical characteristics have been introduced as the novel signal transducers to enhance the detection performance of the clustered regularly interspaced short palindromic repeat‐associated protein (CRISPR‐Cas)‐based nanosensors for cancer diagnostics, exploiting colorimetry, fluorescence, electrochemistry, electrochemiluminescence, and other optical methods.^[^ ^134^ ^]^

**Figure 15:**
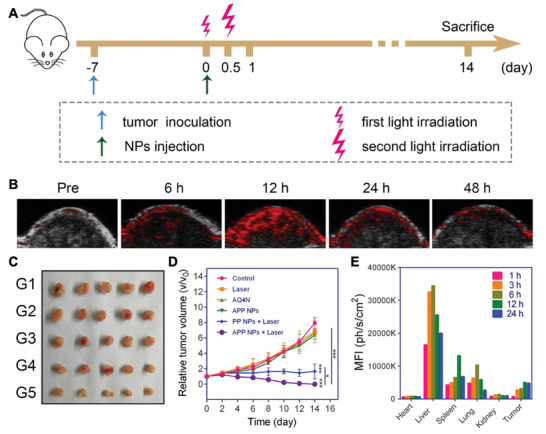
Therapeutic nanoparticles. Reproduced under the terms of the CC‐BY licence.^[^ ^176^ ^]^ Copyrights 2023, the Authors. Published by Springer Nature. A) Experimental procedure of antitumor study in vivo. B) In vivo PA images of tumors after intravenous administration of nanoparticles at different time intervals. C) Photographs of representative tumors dissected from various groups as indicated, G1: PBS, G2: Laser, G3: APP NPs, G4: AQ4N, G5: PP NPs + Laser, G6: APP NPs + Laser. D) Tumor growth curves of mice after different treatments (*n*  =  5). E) Fluorescence intensities of main organs and tumors after injection of APP NPs (*n*  =  3).

Photochemotherapy mediated by nanoparticles is also an emerging research field. In a recent work, PCN‐224 was functionalized with dopamine monomer and loaded with the prodrug banoxantrone (AQ4N). Two consecutive irradiations are performed to promote cellular internalization by photochemical reaction, followed by a second stage where nanoparticles produced reactive oxygen species (ROS) by photothermal conversion. This results in photothermal therapy (PTT) and photodynamic therapy (PDT), which aggravates tumor hypoxia levels and further activate the cytotoxicity of AQ4N for chemotherapy. Both in vitro and in vivo studies produced enhanced anticancer effects compared to traditional chemotherapy (**Figure** **15**).^[^ ^176^ ^]^

### 8.2 Electroceuticals

**Figure 16:**
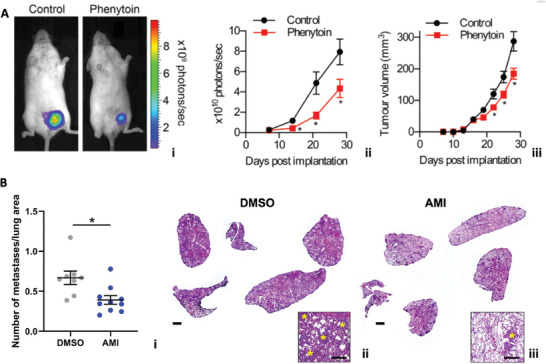
Electroceuticals: small molecules modulators which can alter the electrical state of a cell or tissue by acting on the inhibition of specific players. A) In vivo evidence showing suppression of breast cancer metastasis in mice by blocking VGSC activity with phenytoin. (i) Bioluminescent images of control and phenytoin‐treated mice, 4 weeks after implantation; (ii) Bioluminescence measured from primary tumors post‐implantation; (iii) Volume derived from caliper measurement of primary tumors over the same periods. Reproduced under the terms of the CC‐BY licence.^[^ ^120^ ^]^ Copyrights 2019, the Authors. Published by MDPI. B) Amiodarone treatment depolarizes TNBC RMP resulting in decreased cell migration. i) Number of MDA‐MB‐231 derived metastases per area of lung after daily injections of amiodarone or DMSO for 24 days. (ii) Representative lung tissue sections stained with H&E from animals treated with DMSO or (iii) amiodarone. Scale bar = 1 mm. Inset scale bar = 150 µm. Yellow asterisks indicate metastases. Reproduced with permission from.^[^ ^188^ ^]^ Copyrights 2021, Elsevier.

The emerging field of bioelectricity has revealed numerous new roles for ion channels beyond the nervous system, which can be exploited for applications in regenerative medicine.^[^ ^120^ ^]^ Developing such biomedical interventions for birth defects, cancer, traumatic injury, and bioengineering first requires knowledge of ion channel targets expressed in tissues of interest. This information can then be used to select combinations of small molecule inhibitors and/or activators that manipulate the bioelectric state.^[^ ^186^ ^]^ For example, blocking voltage‐gated sodium channels (VGSC) activity was found to reduce tumor size in mice (**Figure** **16**A).^[^ ^120^ ^]^ Here, metastatic MDA‐MB‐231 cells were injected into female immunodeficient mice and the involvement of a VGCS called Nav1.5 in tumor progression was monitored in vivo using lentiviral shRNA.^[^ ^187^ ^]^ Nav1.5 slowed primary tumor growth, reduced MMP9 expression, increased apoptosis, inhibited local invasion and metastasis to the liver, lungs, and spleen. Triple‐negative breast cancer (TNBC) progression was also inhibited with electroceuticals that blocked K^[+]^ channels, found overexpressed in breast cancer (Figure 16B).^[^ ^188^ ^]^ The induced V_m_ alteration suggested that this route could be a new strategy that targets the cadherin‐11 and MAPK pathway to target TNBC.

#### 8.2.1 Tumor Treating Fields (TTFs)

Tumor Treating Fields (TTFs) are a class of novel non‐invasive treatments for solid tumors that uses low‐intensity alternating electric fields to disrupt cell division and inhibit cancer cell growth.^[^ ^20^ ^]^ It does not involve the use of nanoparticles or nanostructures, but it is mentioned in this review due to its relevance in the state‐of‐the‐art methods aimed at treating cancer by exploiting the electrical properties of cancerous cells and tissues. Electric fields interfere with the alignment of the microtubules during cell division, causing the cells to undergo apoptosis.^[^ ^17^ ^]^ In addition, TTFs can also prevent cells from entering the S phase of the cell cycle, which is essential for cell growth and proliferation. TTFs have been approved by the US Food and Drug Administration (FDA) for the treatment of recurrent glioblastoma and newly diagnosed glioblastoma.^[^ ^21^, ^189^ ^]^ Ongoing clinical trials are showing promising results also in the treatment of non‐small cell lung cancer, pancreatic cancer, and ovarian cancer.^[^ ^17^, ^23^ ^]^ TTFs can be delivered using a portable medical device called Optune, consisting of a set of insulated electrodes placed on the patient's scalp.^[^ ^22^ ^]^ Combination therapies have also attracted interest as a method to enhance TTFs efficacy, for example by combination with immunotherapy.^[^ ^190^ ^]^ Evidence suggests that low frequency magnetic fields (LF‐MF) suppressed tumor growth and influenced the function of immune system.^[^ ^191^ ^]^ However, the mechanisms behind the effect of LF‐MF remain unclear.^[^ ^192^, ^193^ ^]^

**Figure 17:**
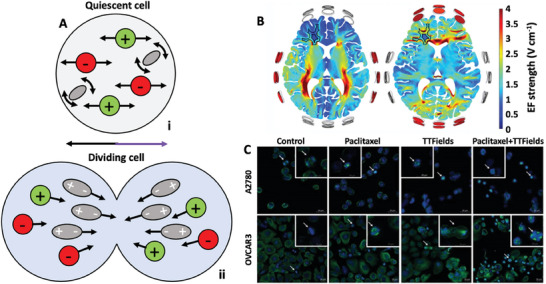
Tumor treating fields have demonstrated anticancer effects. A) AC field distribution in quiescent (i) and dividing (ii) cells. Readapted with permission from.^[^ ^17^ ^]^ Copyrights 2007, National Academy of Science. B) Colormaps depicting the maximum TTFs intensity distributions in default layouts for brain tissue of a patient with pairs of transducer arrays positioned left‐right (left) and anterior‐posterior (right) in axial slices through the brain. Reproduced with permission from.^[^ ^17^ ^]^ Copyrights 2007, National Academy of Science. C) Confocal fluorescence microscopy in A2780 cells for 8 h and OVCAR‐3 cells for 16 hr. The small micrographs represent the cell phases of metaphase and late anaphase. Green: tubulin; blue: DAPI‐stained DNA. Reproduced under the terms of the CC‐BY licence.^[^ ^20^ ^]^ Copyrights 2016, the Authors. Published by Wiley and Sons.

Tumor‐bearing mice carrying lung cancer were exposed to a LF‐MF (0.4T, 7.5 Hz) for 35 days, resulting in tumor growth inhibition. LF‐MF were reported to block cell growth in vitro by stabilization of the p53 protein, which in turn inhibited cell iron metabolism and enhanced miR‐34a transcription.^[^ ^191^ ^]^ **Figure** **17**A displays AC electric fields distribution in quiescent and in dividing cells. Inside quiescent cells the field is uniform, and the oscillating electric forces result only in vibration of ions and dipoles.^[^ ^191^ ^]^ In contrast, the nonuniform field within dividing cells induces forces pushing all dipoles toward the furrow.^[^ ^191^ ^]^ TTFs were demonstrated to be successful in aiding the treatment of glioblastoma.^[^ ^189^ ^]^ These findings led to the initiation of a pilot clinical trial of the effects of TTFs in 10 patients with recurrent glioblastoma (GBM), yielding to doubled medians of survival associated to control patients.^[^ ^17^ ^]^ In a recent study, field strength within a tumor did not correlate with its size and shape. Instead, it was found higher in superficial tumors (Figure 17B). Compared with a default layout, the largest increase in field strength was 184%, and the highest average field strength induced in a tumor was 2.21 V cm^−1^.^[^ ^21^ ^]^ A combination of TTFs and chemotherapy in a different study was used to treat a newly diagnosed glioblastoma patient with high efficiency compared to temozolomide chemotherapy alone. Cognitive screening was improved in response to temozolomide and TTF at 150 kHz. The effect of TTFs combined with chemotherapy was studied on ovarian cancer cells (TTFs at 175 kHz and paclitaxel for 72 h).^[^ ^20^ ^]^ The cells exhibited a significant reduction in cell viability of more than 44.6% of the total cell count and a reduction in clonogenic potential (23.8%) in the whole‐cell count. These cell numbers were significantly reduced in comparison to the negative control and blank cells, as shown in Figure 17C.

## 9 Conclusions and Outlook

The relevance of bioelectrical cues in living systems, from cells to tissues and entire organisms, has attracted interest since the first half of the 20th century. In the context of cancer, it is now well assessed that cancerous cells exhibit characteristic electrical properties given by the altered ion flux across the cell membrane. The intuition that this effect would be mirrored at the macroscopic level has been further confirmed with experimental evaluations that involved studies of cancer cell populations, and electrophysiological recording and stimulation of tumoral masses. However, the technological bottleneck makes progress slow toward the in‐depth understanding of these phenomena. Recording electrophysiological signals from non‐excitable cells implies the development of passive, highly sensitive methods that allow for accessing this biological information without altering the electrical properties of the cell, and measuring steady tiny signals for long time is still a challenge. At the same time, the available information on the subject is being exploited at its best with the objective of developing novel diagnostic and therapeutic strategies based on bioelectricity. This field of research is at the forefront of medicine and technology, and further technological improvements in the area of nanoscience are crucial to enabling the understanding of these biological phenomena, that in turn will allow to aim at the design of highly sensitive systems for cancer diagnostics, monitoring, and therapy.

## Conflict of Interest

The authors declare no conflict of interest.
